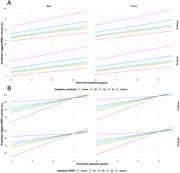# Differences in growth rates and spatial patterns of white matter hyperintensities between beta‐amyloid burden and vascular risk

**DOI:** 10.1002/alz70856_106165

**Published:** 2026-01-07

**Authors:** Jeremy F. Strain, Maryam Rahmani, Chia‐Ling Phuah, Donna Dierker, Jingqin Luo, Christopher J. Owen, Andrei G. Vlassenko, Hussain Jafri, Pierrick Bourgeat, Jurgen Fripp, Liang Jin, Krista L. Moulder, Tammie L.S. Benzinger, Chengjie Xiong, Jin‐Moo Lee, Michael W Weiner, Colin L Masters, John C. Morris, Kyle B. Womack, Manu S. Goyal

**Affiliations:** ^1^ Washington University in St. Louis School of Medicine, St. Louis, MO, USA; ^2^ Washington University in St. Louis School of Medicine, St. Louis, MO, USA; ^3^ Dignity Health dba Barrow Neurological Institute/St. Joseph's Hospital & Medical Center, Phoenix, AZ, USA; ^4^ Washington University School of Medic, St. Louis, MO, USA; ^5^ Department of Surgery, Washington University, St. Louis, MO, USA; ^6^ Washington University School of Medicine, St. Louis, MO, USA; ^7^ Mallinckrodt Institute of Radiology, Washington University School of Medicine, St. Louis, MO, USA; ^8^ Knight Alzheimer Disease Research Center, St. Louis, MO, USA; ^9^ Hope Center for Neurological Disorders, St. Louis, MO, USA; ^10^ The Australian e‐Health Research Centre, CSIRO, Brisbane, QLD, Australia; ^11^ Florey Institute of Neuroscience and Mental Health, Parkville, VIC, Australia; ^12^ The Charles F. and Joanne Knight Alzheimer Disease Research Center, St. Louis, MO, USA; ^13^ Knight Alzheimer's Disease Research Center, St. Louis, MO, USA; ^14^ Hope Center for Neurological Disorders, Washington University School of Medicine, St. Louis, MO, USA; ^15^ Washington University in St. Louis, St. Louis, MO, USA; ^16^ Washington University School of Medicine in St. Louis, St. Louis, MO, USA; ^17^ University of California San Francisco (UCSF), San Francisco, CA, USA; ^18^ The Florey Institute of Neuroscience and Mental Health, The University of Melbourne, Parkville, Melbourne, VIC, Australia; ^19^ Knight Alzheimer Disease Research Center, Washington University School of Medicine, St. Louis, MO, USA; ^20^ Washington University in St. Louis, School of Medicine, St. Louis, MO, USA

## Abstract

**Background:**

There is increasing evidence for an association between white matter hyperintensities (WMH) and brain beta‐amyloid deposition. WMH are not exclusive correlates of a single etiology, and the spatial topography can associate with different pathologic markers of vascular or neurodegenerative disease. How WMH are longitudinally associated with brain beta‐amyloid burden requires further investigation, particularly with respect to co‐existent vascular risk factors and differences across brain regions.

**Method:**

We retrospectively measured WMH on MRI and vascular risk factors in a combined neuroimaging data set comprised of the ADNI, AIBL and OASIS3 studies, which included harmonized centiloid estimates of beta‐amyloid burden from PET imaging. WMH were measured using the TrUE‐Net algorithm. Vascular risk factors were extracted from provided clinical data and used to calculate individual revised Framingham Stroke Risk Profile (FSRP) scores. Five established data‐driven WM regions (juxtacortical, deep frontal, periventricular, parietal, posterior) were used for regional relationships with WMH volume and growth. Linear mixed effects modelling was used to determine the relationship between the growth rate of normalized regional WMH volumes and baseline beta‐amyloid burden, controlling for age, sex, APOE4 status, and vascular risk factors.

**Result:**

1245 participants [48.7% female, mean age 71.7 y (SD 7.6 y)] had at least 3 brain MRIs suitable for WMH volume measurement. Linear mixed models demonstrate robust independent cross‐sectional relationships between WMH and baseline beta‐amyloid burden (*p* <0.001), age (*p* <0.001) and FSRP (*p* <0.001). Growth rates of WMH increased with baseline beta‐amyloid burden (*p* <0.05) and decreased with vascular risk (*p* <0.001), above and beyond age, sex, and APOE4 status. Regional analyses revealed both baseline beta‐amyloid burden and FSRP associated with the juxtacortical deep frontal and periventricular regions, but the parietal region was unique to beta‐amyloid (*p* <0.05). Longitudinally, the association for beta‐amyloid burden (*p* <0.005) persisted in only the parietal WMH and no normalized regional values associated longitudinally with FSRP.

**Conclusion:**

Our study suggests that in Alzheimer disease research cohorts, WMH progression is associated with beta‐amyloid burden, particularly in parietal white matter. Vascular risk associated WMH were influential for WMH volume but did not associate with WMH progression in a regionally specific manner.